# Iota-carrageenan neutralizes SARS-CoV-2 and inhibits viral replication in vitro

**DOI:** 10.1371/journal.pone.0237480

**Published:** 2021-02-17

**Authors:** Martina Morokutti-Kurz, Maria Fröba, Philipp Graf, Maximilian Große, Andreas Grassauer, Janina Auth, Ulrich Schubert, Eva Prieschl-Grassauer

**Affiliations:** 1 Marinomed Biotech AG, Korneuburg, Austria; 2 Institute of Virology, Friedrich-Alexander University Erlangen-Nürnberg (FAU), Erlangen, Germany; University of Washington, UNITED STATES

## Abstract

In the absence of a vaccine and other effective prophylactic or therapeutic countermeasures the severe acute respiratory syndrome-related coronavirus 2 (SARS-CoV-2) remains a significant public health threat. Attachment and entry of coronaviruses including SARS-CoV-2 is mainly mediated by the spike glycoprotein. Here, we show that iota-carrageenan can inhibit the cell entry of the SARS-CoV-2 spike pseudotyped lentivirus in a dose dependent manner. SARS-CoV-2 spike pseudotyped lentivirus particles were efficiently neutralized with an IC_50_ value of 2.6 μg/ml iota-carrageenan. Experiments with patient isolated wild type SARS-CoV-2 virus showed an inhibition of replication in a similar range. In vitro data on iota-carrageenan against various Rhino- and endemic Coronaviruses showed similar IC_50_ values and translated readily into clinical effectiveness when a nasal spray containing iota-carrageenan demonstrated a reduction of severity and duration of symptoms of common cold caused by various respiratory viruses. Accordingly, our in vitro data on SARS-CoV-2 spike pseudotyped lentivirus and replication competent SARS-CoV-2 suggest that administration of iota-carrageenan may be an effective and safe prophylaxis or treatment for SARS-CoV-2 infections.

## Introduction

The spread of Severe Acute Respiratory Syndrome Coronavirus (SARS-CoV-2) around the world has created a pandemic situation regarded as a significant public health threat [1–4]. SARS-CoV-2 is a betacoronavirus closely related to SARS-CoV-1. Although the sequence of the spike glycoprotein (SGP) of SARS-CoV-2 is significantly different compared to the SGP of SARS-CoV-1, both bind to the same receptor, human angiotensin converting enzyme 2 (hACE2). Upon binding to hACE2 the protease TMPRSS2 modifies SGP, thus allowing envelope fusion and viral entry [5]. In addition to this rather specific mechanism, many viruses (including some betacoronaviruses) use cellular polysaccharides as cellular attachment co-receptors, allowing the virus to adhere to the surface of the cell. This unspecific interaction increases the local concentration of viral particles leading to higher infection rates.

All respiratory viruses have in common that they must pass through the physical barrier of mucus and respiratory fluid to reach the cell surface. It is hypothesized that the viruses utilize their positive electrical charge to reach the negatively charged cell surface [6]. Polyanionic molecules such as iota-carrageenan may present a way of trapping the viruses as they move towards the surface of the cell. This current knowledge is backed by a series of experiments showing that direct interaction between virus and Carrageenan is needed to efficiently inhibit infection of cells (Grassauer et al, 2008, Leibbrandt et al. 2010, Graf et al, 2017). The activity of carrageenan is based on its ability to neutralize virus particles when they first enter the nasal cavity, and also when newly synthesized virus particles are released from infected cells. The lack of any pharmacological, immunological, or toxicological activity of large polyanionic molecules such as iota-carrageenan and their lack of absorption or metabolism makes them a safe topical antiviral treatment.

The antiviral activity of iota-carrageenan has been demonstrated in vitro against a variety of respiratory viruses [7–9]. The transferability of these in vitro data into clinical effectiveness was evaluated in four clinical trials [10–14].

The efficacy and safety of an iota-carrageenan containing nasal spray have been studied in more than 600 children and adults suffering from early symptoms of common cold. In all trials a significant reduction of viral load of different respiratory viruses has been demonstrated in the iota-carrageenan treatment group compared to placebo thereby convincingly bridging in vitro neutralization data and effectiveness against a respiratory infection of humans. Additionally, this titer reduction also manifested clinically by reducing the severity and duration of symptoms as well as the number of relapses in the verum group. There were no differences with respect to safety between iota-carrageenan treated patients compared to placebo [6].

Due to the highly transmissible and pathogenic nature of SARS-CoV-2, handling of live virus requires biosafety level 3 (BSL3) containment. Hence, we utilized a recently developed high titer SARS-CoV-2 Spike Pseudotyped Lentivirus (SSPL) to screen potential inhibitors in our BSL2 laboratory. Attachment and entry can be measured via luciferase reporter activity which correlates directly with the efficiency of transduction. Using this lentiviral system, we tested the ability of various sulfated polysaccharides to inhibit viral attachment and entry. We further investigated the antiviral activity of the most promising candidate, iota-carrageenan, against wild type (wt) SARS-SoV-2 in Vero cells.

We discuss implications of the findings for both SARS-CoV-2 countermeasures and potential clinical applications.

## Materials and methods

### SARS-CoV-2 Spike Pseudotyped Lentivirus (SSPL)

Pseudotyped particles were obtained from BPS Bioscience, San Diego CA92121 Catalog#: 79942. The pseudovirions contain SARS-CoV-2 Spike protein (Genbank Accession #QHD43416.1) and the firefly luciferase gene driven by a CMV promoter. Therefore, the spike-mediated cell entry can be measured via luciferase reporter activity. The SARS-CoV-2 Spike pseudotyped lentivirus has been designed to measure the activity of neutralizing antibody against SARS-CoV-2 in a BSL2 facility.

### Neutralization test

The pseudotyped virus was used according to the manufacturer´s instructions: Approximately 7500 ACE2-HEK293 cells/well were infected with 750 infectious particles (MOI = 0.1) of SARS-CoV-2 Spike pseudotyped lentivirus (Luc reporter). Virus was incubated with buffer (controls) or the tests substances for 30 minutes before infection (designated as concentration “virus contact”). For infection, 5 volumes of cell culture medium (MEM (Merck KGA, Germany) containing 10% FCS (Merck KGA, Germany), 4 mM glutamine (Merck KGA, Germany), 1 mM sodium pyruvate (Merck KGA, Germany), and 1% penicillin/streptomycin (Merck KGA, Germany) were added, resulting in 1:6 dilution of the initial concentration of the test substance. (24 hours after infection, the medium was changed to fresh cell culture medium. 48 hours after infection plates were lysed by freeze/thaw before luciferase reagent (Bright Glow, Promega, Madison, WI) was added to cells to measure the luciferase activity using a BMG Fluostar Microplate reader. Mock-infected cells and infected, mock-treated (0.5% NaCl) cells served as positive and negative control. Luciferase data were routinely corrected with metabolic data (Alamar blue) derived from a parallel plate with identical set-up.

### Human coronavirus OC43 (hCoV-OC43)

hCoV OC43 was obtained from the ATCC and propagated in Vero (embryonic African green monkey kidney) cells that were purchased from the ATCC. The cells were cultivated in OptiPro serum free medium (Life Technologies Thermo Scientific, Waltham, USA supplemented with 4 mM L-glutamine (Merck KGA, Germany). Virus stocks were frozen at −80°C and virus titers were determined by endpoint titration assay.

### Virus reduction assay

The assay was adapted for hCoV OC43 from a protocol described elsewhere [9]. In short, the virus was preincubated with a semilogarithmic dilution series of polymers control solutions for 30 minutes before it was added to a monolayer of Vero cells for infection. After an infection period of 45 minutes at RT the inoculum was removed; cells were overlaid with medium containing test substance and then cultured at 37°C, hereby maintaining the same concentrations of active agent as in the prophylactic treatment. Staining was performed on fixed cells using an antibody directed against the hCoV-OC43 nucleoprotein as a primary antibody (Merck #MAB9013) followed by a horse radish peroxidase labeled detection antibody (Thermo Scientific #31430) and TMB as substrate. For detection, a BMG Fluostar Microplate reader was utilized. To enable direct comparison of the effectiveness of the test substances, the IC_50_ value of each sample was calculated for a sigmoidal dose–response model with XLfit Excel add-in version 5.3.1.

### SARS-CoV-2PR-1

The virus strain SARS-CoV-2_PR-1_, isolated from a 61 year old patient, was amplified in Vero B4 cells as described in [15]. Viral titers were determined by an endpoint titration assay. For the generation of new virus stock, virus containing cell culture supernatant was harvested 72 hpi and passed through a 0.45 μm pore-size filter. Virus stocks were stored at −80°C until further usage. For Western Blot analysis, Vero B4 cells were infected with SARS-CoV-2_PR-1_ (multiplicity of infection (MOI = 0.02) for 1 h, washed and further treated with interventions. 72 hpi, virus-containing cell culture supernatants were harvested and released virions were purified through 20% (w/v) sucrose cushion (20,000× g, 4°C, 90 min).

For titer determination of SARS-CoV-2_PR-1_ virus stocks Vero B4 cells were infected with serial dilutions of the virus stock over 72 h. Afterwards cells were fixed (4% PFA), permeabilized (0.5% Triton/PBS), blocked (1% BSA/PBS-T) and finally stained with a SARS-CoV-2 NP antibody (Biozol). Endpoint of virus infection was analyzed via fluorescence microscopy and viral titer was calculated by the method of Reed and Muench [16].

### Virus reduction assay

Vero B4 cells were maintained in Dulbecco’s Modified Eagle’s Medium (DMEM) containing 10% (v/v) inactivated fetal calf serum (FCS), 2 mM l-glutamine, 100 U/mL penicillin, and 100 μg/mL streptomycin. Confluent monolayers of Vero B4 cells were infected in FCS free DMEM with the field isolate SARS-CoV-2PR1 at an MOI of 0.02 for 1 hour. In order to remove input virus cells were washed and afterwards treated with interventions. 72 hours post infection, cells were lysed in radio immunoprecipitation assay (RIPA) buffer (150 mM NaCl, 50 mM Tris-HCl pH 8.0, 1% NP-40, 0.5% Na-deoxycholate, 0.1% sodium dodecyl sulfate (SDS), 10 mM ethylenediaminetetraacetic acid (EDTA)) containing protease inhibitor cocktail Complete (Roche, Basel, Switzerland), 5 mM N-ethylmaleimide (NEM), and 1 mM phenylmethylsulfonylfluoride (PMSF) and further used for Western Blot analysis.

### SDS-PAGE and Western blotting

Protein samples were separated by SDS-PAGE, transferred onto nitrocellulose membranes, blocked with 3% bovine serum albumin and incubated with the appropriate primary antibody (Ab). Viral proteins were detected by antibodies derived from convalescent SARS-CoV-2 patient sera. The anti-human secondary antibody coupled to horseradish peroxidase (HRP) was obtained from Dianova (Hamburg, Germany).

### Assessment of cell viability

Viability of infected and treated cells was assessed by the water-soluble tetrazolium salt (WST)-1 assay (Roche) according to the manufacturer’s instructions.

### Determination of the amount of viral RNA copies from released viruses by qRT-PCR

Virus was quantified by real-time PCR AgPath-ID One-Step RT-PCR Kit from Ambion (Cat: 4387424) software v2.3 (applied Bioscience). PCR primers were used according to [17]: RdRp_fwd: 5′-GTG-ARA-TGG-TCA-TGT-GTG-GCG-G-3′ and RdRp_rev 5′-CAR-ATG-TTA-AAS-ACA-CTA-TTA-GCA-TA-C-3′. Probe was 5′—CAG-GTG-GAA-/ZEN/CCT-CAT-CAG-GAG-ATG-C -3′ (Label: FAM/IBFQ Iowa Black FQ). As positive control a specific target for E and RdRp gen of SARS-CoV2 was used and made by Integrated DNA Technologies. Control: 5´-TAA-TAC-GAC-TCA-CTA-TAG-GGT-ATT-GAG-TGA-AAT-GGT-CAT-GTG-TGG-CGG-TTC-ACT-ATA-TGT-TAA-ACC-AGG-TGG-AAC-CTC-ATC-AGG-AGA-TGC-CAC-AAC-TGC-TTA-TGC-TAA-TAG-TGT-TTT-TAA-CAT-TTG-GAA-GAG-ACA-GGT-ACG-TTA-ATA-GTT-AAT-AGC-GTA-CTT-CTT-TTT-CTT-GCT-TTC-GTG-GTA-TTC-TTG-CTA-GTT-ACA-CTA-GCC-ATC-CTT-ACT-GCG-CTT-CGA-TTG-TGT-GCG-TAC-TGC-TGC-AAT-ATT-GTT-3´

### Inhibitors

Iota-, kappa- and lambda-carrageenan were purchased from Dupont former FMC Biopolymers (both Philadelphia, PA). Fucoidan from Undaria pinnatifolia and Fucus vesiculosis were from Marinova (Marinova Pty Ltd, Australia), Carboxymethylcelluslose (CMC) was from Mare Austria GmbH, Hydroxypropylmethylcellulose (HPMC) from Fagron (Fagron BV, The Netherlands), galactose-4-sulfate was purchased from Merck KGA (Germany). The dry polymer powders were dissolved in cell culture water (B Braun Melsungen AG, Germany) to a final concentration of 2.4 mg/ml containing 0.5% NaCl (Merck KGA, Germany). This stock solution was sterile filtered through a 0.22mm filter (Sarstedt, Germany) and stored at 4°C until use.

For testing of wild type of wild type SARS-CoV-2 virus a commercially available nasal spray containing 1.2 mg/ml iota-carrageenan was used.

### NMR analysis of kappa- and lambda-carrageenan

10 mg kappa and lambda-carrageenan samples were sent to Spectral Services, Köln, for NMR measurements. In brief, 10 mg substance were dissolved in 1 ml D_2_O containing 3-(trimethylsilyl)propionic acid-d4 sodium salt (0.01% as standard). Measurements for ^1^H spectra were done with an Avance III HD 500 MHz NMR spectrometer (Bruker, Billarica, MA).

## Results

### Iota-carrageenan neutralizes SARS-CoV-2 Spike Pseudotyped Lentivirus (SSPL) particles

**Fig 1:**
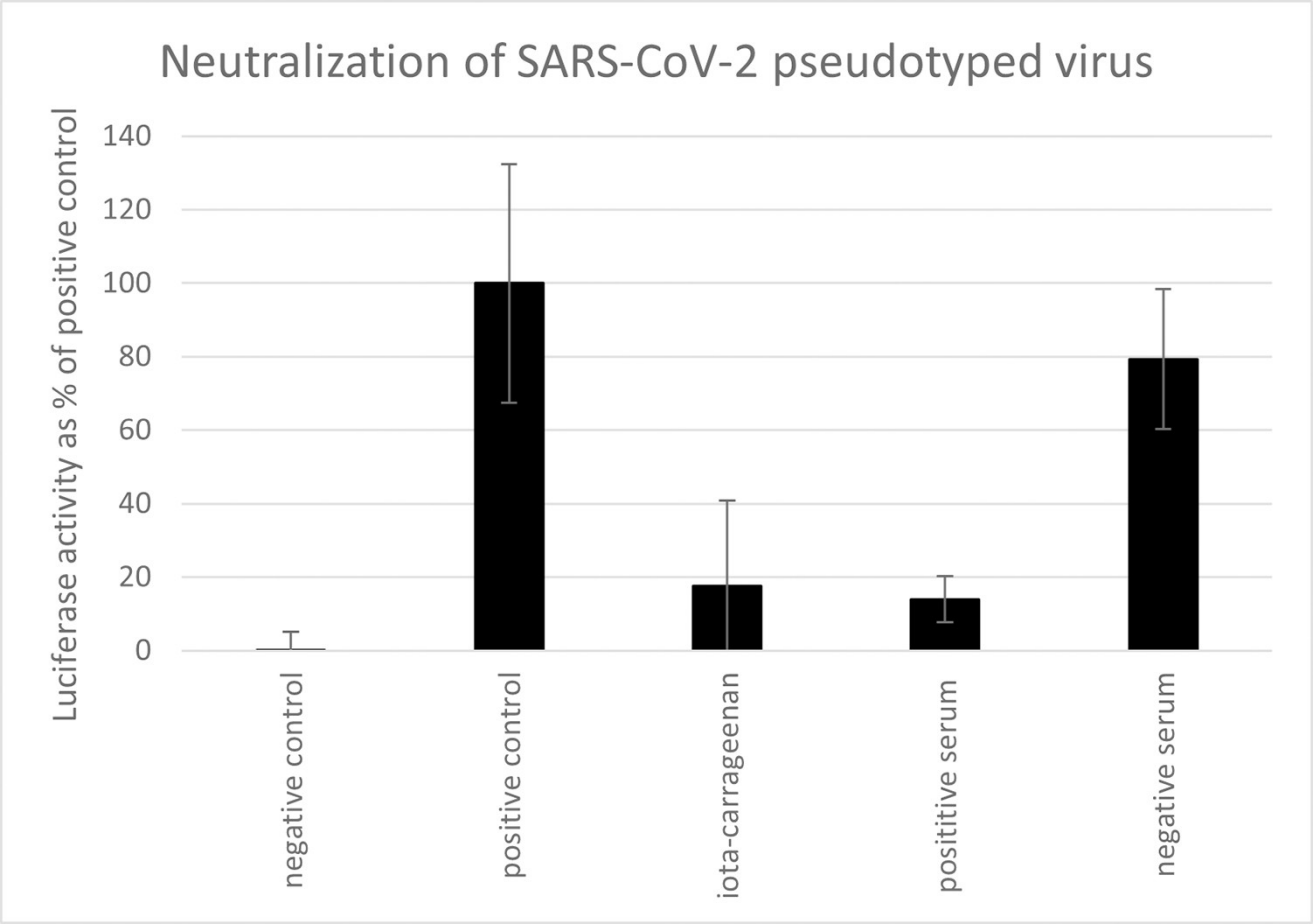
Neutralization of SARS-CoV-2 pseudotyped lentivirus. Neutralization assay with iota-carrageenan compared to neutralizing serum and negative serum. Approximately 7500 ACE2-HEK293 cells/well were infected with SARS-CoV-2 Spike pseudotyped lentivirus at an MOI of 0.1 (Luc reporter). Mock infected and mock treated infected cells served as negative control and positive control, respectively. 10 μg/ml iota-carrageenan and serum diluted 1:15 were incubated with the virus before infection. For infection, the virus/polymer or virus/serum inoculum was diluted with 5 volumes of medium. 48 hours after infection the infection efficiency was determined by measuring the luciferase activity. Luciferase data were corrected with metabolic data (Alamar blue) derived from a parallel plate with identical set-up. Data are normalized to % positive control and represent means of triplicates with standard deviation indicated.

To determine whether iota-carrageenan can block the infection of cells with SSPL we incubated the viral particles with 10 μg/ml iota-carrageenan dissolved in 0.5% NaCl solution for 30 minutes prior infection. For infection, this mixture was diluted according to the manufacturer´s protocol by the addition of 5 volumes of medium. Mock-infected cells and infected, mock-treated (0.5% NaCl) cells served as positive and negative control, respectively. In addition, we tested the serum of a patient tested positive for SARS-CoV-2 by PCR, who showed seroconversion in an IgG ELISA specific for SGP and nucleoprotein of SARS-CoV-2. This serum effectively neutralized the virus while a control serum from 2011 had no effect on virus infection (Fig 1). Serum was used in a 1:15 dilution. With 82% neutralization capacity, 10μg/ml iota-carrageenan were equally effective as the 1:15 diluted antiserum (86% neutralization). These results demonstrate that low concentrations iota-carrageenan are capable of neutralizing SSPL particles.

#### Neutralization of SSPL particles with iota-carrageenan is dose dependent

**Fig 2:**
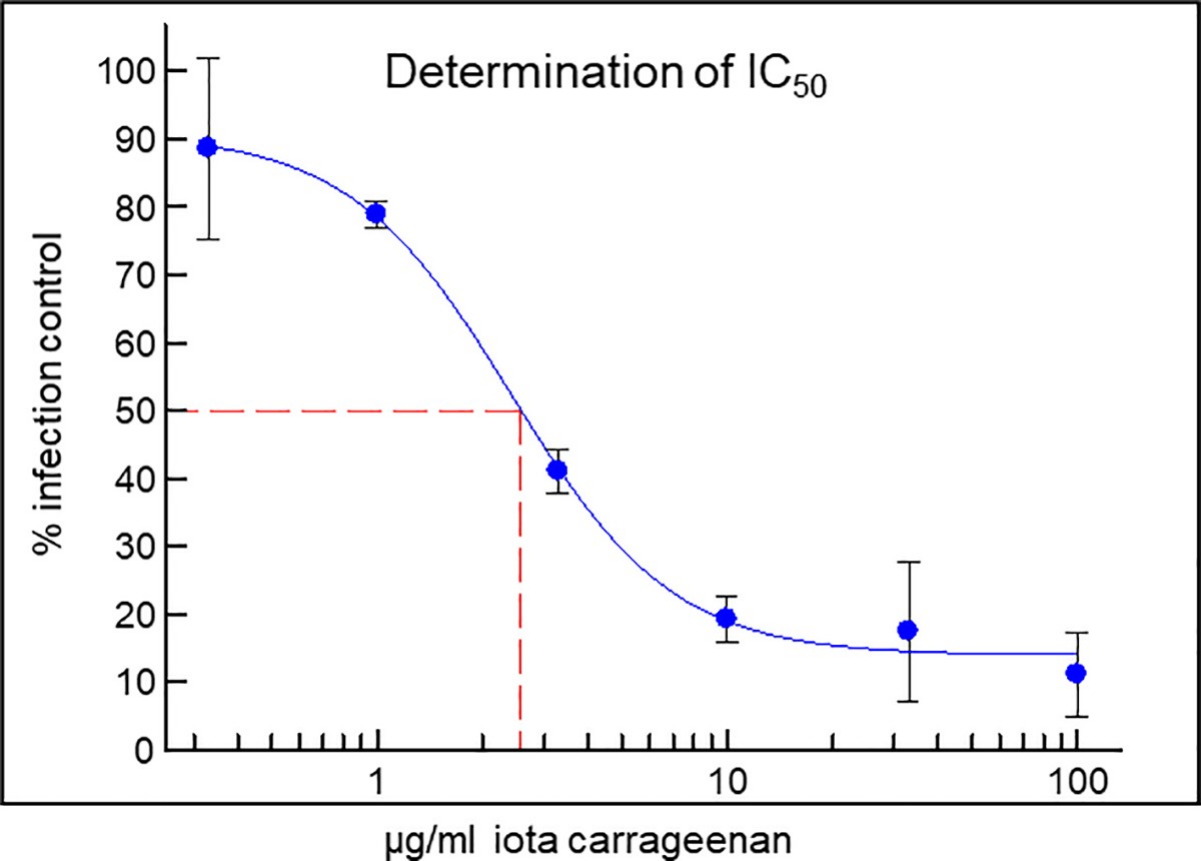
Determination of IC_50_. Titration of iota-carrageenan (100 μg/ml to 0.33 μg/ml) and determination of IC_50_. ACE2-HEK293cells/well were infected with SARS-CoV-2 Spike pseudotyped lentivirus at an MOI of 0.1 (Luc reporter). Mock infected and mock treated infected cells served as negative control (0% infection) and positive control (100% infection control), respectively. The virus was incubated with different concentrations of iota-carrageenan for 30 minutes before infection. 48 hours after infection the infection efficiency was determined by measuring the luciferase activity. Luciferase data were corrected with metabolic data (Alamar blue) derived from a parallel plate with identical set-up. Data represent means of triplicates ± standard deviation. The red line indicates the determination of the IC_50_.

As shown in Fig 2 the neutralization activity of iota-carrageenan is dose dependent with and IC_50_ of 2.6 μg/ml. Iota-carrageenan concentrations of 10 μg/ml or higher resulted in a reduction of the signal by more than 80% indicating a plateau above this concentration. Interestingly, the presence of only 1 μg/ml iota-carrageenan resulted in a detectable reduction of infectivity by more than 20%.

#### Comparison of iota-carrageenan with other sulfated and non-sulfated polymers as well as a low molecular weight sulfated sugar

**Fig 3:**
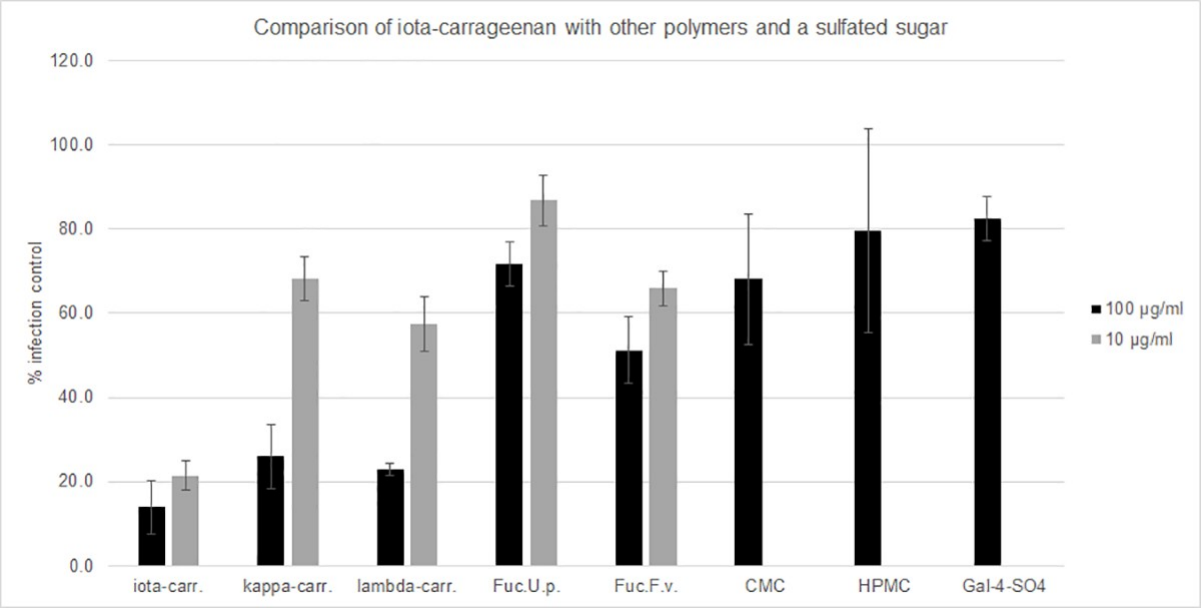
Comparison of iota-carrageenan with other polymers and a sulfated sugar. Neutralization assay with iota-carrageenan compared to other sulfated and non-sulfated polymers as well as a sulfated sugar. ACE2-HEK293cells/well were infected with SARS-CoV-2 Spike pseudotyped lentivirus (Luc reporter). Mock infected and mock treated infected cells served as negative control (0% infection) and positive control (100% infection control; y-axis), respectively. 100 μg/ml and 10 μg/ml sulfated polymers and 100 μg/ml non-sulfated polymers and galactose-4-sulfate were incubated with the virus for 30 minutes before infection. Infection efficiency was determined by measuring the luciferase activity 48 hours post infection. Luciferase data were corrected with metabolic data (Alamar blue) derived from a parallel plate with identical set-up. Data represent means of triplicates ± standard deviation. Abbr.: Iota-carr. (iota-carrageenan), kappa-carr. (kappa-carrageenan), lambda-carr, (lambda-carrageenan), Fuc.U.p. (high molecular weight fucoidan from Undaria pinnatifida), Fuc.F.v. (high molecular weight fucoidan from Fucus vesiculosus), CMC (carboxymethylcellulose), HPMC (hydroxypropylmethylcellulose), and Gal-4-SO4 (galacatose-4-sulfate).

Several polymers were tested for their ability to neutralize SSPL particles (Fig 3). While iota-carrageenan effectively inhibited the virus at 10 μg/ml, kappa-carrageenan and lambda-carrageenan were only active at 100 μg/ml. High molecular weight fucoidan from two different species, Undaria pinnatifida and Fucus vesiculosus, resulted in less than 50% reduction of infection at the higher concentration (100 μg/ml). Polymers without sulfate groups, carboxymethylcellulose (CMC) and hydroxypropylmethylcellulose (HPMC) were inactive in this assay. Also, a low molecular weight sulfated sugar, galactose-4-sulfate did not neutralize SSPL.

The composition of the kappa- and lambda-products was determined by NMR analysis to evaluate if iota-carrageenan is present in these preparations. Surprisingly, kappa-carrageenan contained 16%, and lambda-carrageenan 27.3% iota-carrageenan (Table 1).

**Table 1:** Results of NMR analysis of kappa- and lambda-carrageenan preparations.

Carrageenan	Lambda	Nu	Iota	Kappa
**Lambda-carrageenan**	19,6	10,0	27,3	41,8
**Kappa-carrageenan**	-	0,4	16,0	83,6

10 mg of the respective substance were dissolved in 1 ml D_2_O containing 3-(trimethylsilyl)propionic acid-d4 sodium salt (0.01% as standard). Measurements for ^1^H spectra were done with an Avance III HD 500 MHz NMR spectrometer (Bruker, Billarica, MA). Data were normalized to the total carrageenan content.

### Iota-carrageenan shows antiviral activity SARS-CoV-2

**Fig 4:**
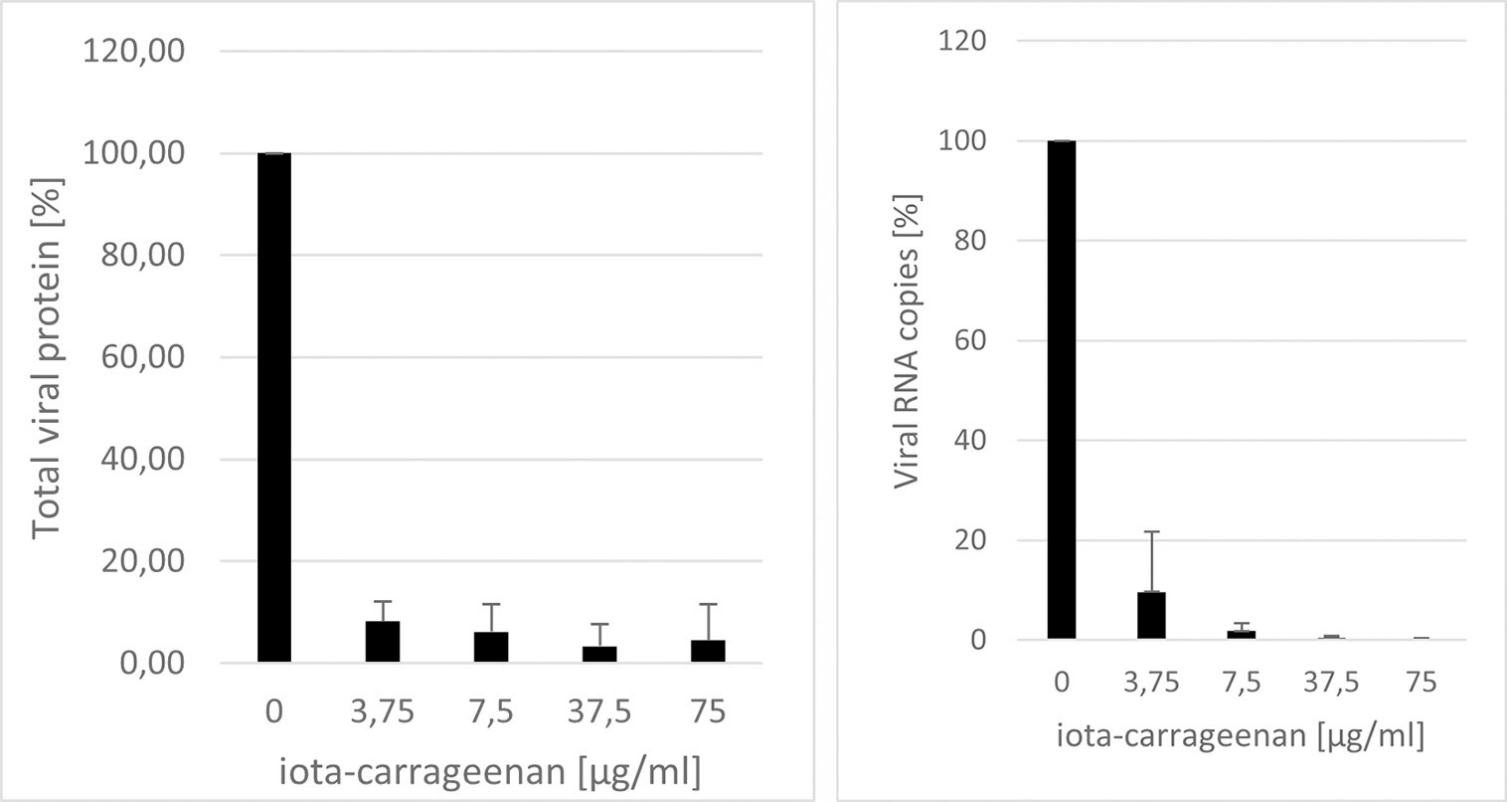
Iota-carrageenan restricts replication of SARS-CoV-2 in Vero B4 cells. (A): Western blot analysis of virus fractions (NP = Nucleoprotein) after treatment with increasing concentrations of Carrageenan for 3 days. Vero B4 cells were infected with SARS-CoV-2 at a MOI of 0.2. 1 hour after infection and subsequent removal of input virus, cells were treated with different concentrations of carrageenan. After 3 days, cells were harvested and virions from three independent experiments were purified and analyzed by Western blot using SARS-CoV-2 convalescent serum. Densitometric analyses of Western blots were performed using AIDA®. Bars represent mean values of three independent experiments, ±standard deviation. (B): Real time PCR analysis of purified virions. The experiment was performed as described in Fig 4A. The purified virions were analyzed using real time PCR. Shown are the results of three independent experiments ±standard deviation.

In order to determine whether the results obtained SSPL also apply to SARS-CoV-2 virus, Vero B4 cells were infected with SARS-CoV-2_PR-1_. One hour post infection, the input virus was removed and dilutions of the nasal spray were added to the cells. Three days post infection (dpi) cell culture supernatants were harvested and virus production was analyzed by Western blot (Fig 4A) or quantitative PCR (Fig 4B). As expected, treatment with carrageenan led to a strong reduction of SARS-CoV-2 replication. At the lowest concentration of 3.75 μM iota-carrageenan the production of progeny virions was almost completely blocked.

### Carrageenan does not show any cytotoxicity on Vero B4 cells

**Fig 5:**
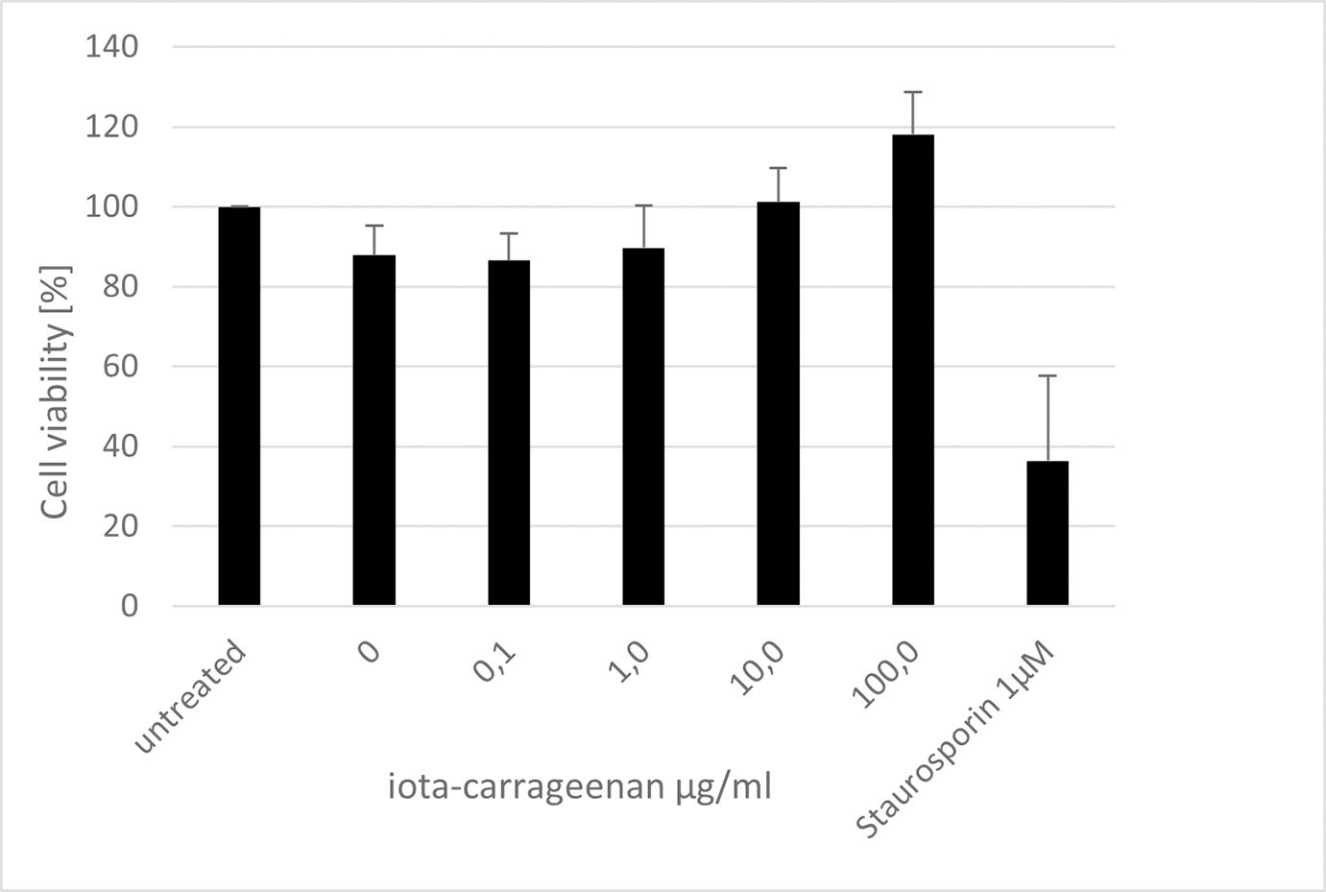
Influence of carrageenan on the cell viability of Vero B4 cells. Following treatment with different concentrations of carrageenan (iota-carrageenan concentrations are indicated at the x-axis) for three days, the influence on cell viability was measured by water-soluble tetrazolium salt (WST)-1 assays. Bars represent mean values of 3 independent experiments ±SD. Staurosporin was used as a positive control.

To control for potential unspecific effects of carrageenan treatment on cell viability, water-soluble tetrazolium salt (WST)-1 assays were performed in uninfected Vero B4 under otherwise identical conditions. The results show that treatment at concentrations which effectively suppress SARS-CoV-2 replication have no impact on cell viability (Fig 5). Moreover, theTD50 could not be determined as a 50% reduction of cell survival was not reached even under the highest concentration tested. Staurosporine, a known inducer of apoptosis, was used as a positive control at a concentration of 1 μM.

### Comparison of SARS-CoV-2 and hCoV OC43 concerning their sensitivity to iota- and kappa-carrageenan

Both, SARS-CoV-2 and hCoV OC43, belong to the genus of beta-coronaviridae. While SARS-CoV-2 newly emerged in late 2019, hCoV OC43 was identified in 1967 [18]. Infection with hCoV OC43 usually leads to common cold symptoms and only rare cases of severe infection result in viral pneumonia. Coronaviruses are commonly detected in patients suffering from common cold and usually represent 10–15% of the cases. In clinical studies with an iota-carrageenan containing nasal spray more than 30% of the tested subjects that were virus-positive had an infection with either hCoV OC43 or 229E (alpha-coronavirus) with an equal 50:50 split between those two viral subtypes. In these clinical trials effectiveness against coronaviruses was clearly demonstrated. Therefore, we wanted to compare the in vitro neutralisation capacity of iota- and kappa-carrageenan against clinically sensitive hCoV OC43 with the one against SSPL. A cell culture assay with hCoV OC43 was established and the effect of iota-carrageenan, kappa-carrageenan and Carboxymethylcellulose (CMC) on viral replication was tested. The polymers were titrated from 100 μg/ml to 0.007 μg/ml. Iota-carrageenan dose-dependently inhibited hCoV OC43 replication with an IC_50_ value of 0.33 μg/ml, while 100 μg/ml kappa-carrageenan resulted in an inhibition of less than 50%. Similarly, a more than 90% reduction of SARS-CoV-2 replication was reached at an iota-carrageenan concentration of 3.5 μg/ml while the control polymer CMC did not show any inhibition. These data demonstrate that SSPLs and wt SARS-CoV-2 are similarly sensitive to iota-carrageenan as hCoV OC43 (Table 2).

**Table 2:** Comparison of IC_50_ values of iota-carrageenan for SSPL and CoV OC43.

Virus strain	Iota-carrageenan IC_50_ [μg/ml]
SSPL	2.58 (1.68–3.48)
hCoV OC43	0.33 (0.01–0.65)
SARS-CoV-2 (western blot)	0.046 (-0.09–0.18)
SARS-CoV-2 (PCR)	1.54 (1.05–2.03)

IC_50_ of iota-carrageenan against SSPL, SARS-CoV-2 and hCoV OC43. 95% confidence interval (CI) is given in brackets.

## Discussion

In this study we utilized a SARS-CoV-2 Spike Pseudotyped Lentivirus to test the neutralization efficacy of different sulfated polymers. An analogous assay was recently used to test the neutralization potential of serum IgG during the active and convalescent phase of COVID-19. While both, asymptomatic patients as well as symptomatic patients, had neutralizing serum IgG antibodies, also in both groups a reduction of the antibody levels and neutralization capacity was observed within 2 to 3 months after infection. The authors speculate that the observed decrease of antibodies and neutralization capacity may pose a risk for using an “immunity passport” and that prolongation of public health interventions may be needed [19]. The findings are also important with respect to the development of a vaccination against SARS-CoV-2. Therefore, the formation of long-lasting neutralizing antibodies and their protective potential will be key factors for success. In the light of these findings the neutralization capacity of iota-carrageenan, which was in the same range as a positive serum from a COVID-19 patient and in the same range as soluble ACE2 receptor protein (IC_50_ ~ 0.5–2 μg/ml; [20]), may be an important additional option for prophylactic and therapeutic intervention.

A comparison of iota-carrageenan with other sulfated polymers such as kappa- and lambda-carrageenan and fucoidans from different species showed that iota-carrageenan has superior efficacy. While 100 μg/ml of kappa- and lambda-carrageenan also neutralized SARS-CoV-2 effectively, the fucoidans were practically ineffective. NMR analysis of kappa- and lambda-carrageenan revealed surprisingly high amounts of iota-carrageenan, namely 16% and 27% in the kappa- and lambda-products, respectively. At 10 μg/ml iota-carrageenan showed a neutralization capacity of 79%. With the 100 μg/ml kappa- and lambda-preparations a similar neutralization of approximately 80% was reached. As the iota-carrageenan concentration in these preparations is 16 and 27 μg/ml, respectively, we hypothesize that the inhibition observed with kappa- and lambda-carrageenan is mainly due to the presence of iota-carrageenan. The inevitable heterogenous nature of carrageenans hampers a deeper understanding about structure-activity relations. More research with iota-carrageenan-free, better refined kappa- and lambda-polymers is needed to allow conclusions on their individual virus blocking effect. The data further highlight the need for quality control via NMR when natural biopolymers are studied.

With an IC_50_ of around 1μg/ml, wt SARS-CoV-2 was similarly sensitive to iota-carrageenan as other respiratory viruses for which antiviral effectiveness has been established in cell culture. Human rhinovirus titers were reduced by more than one log when 5 μg/ml iota-carrageenan were added before and during the infection [7]. The IC_50_ values for the inhibition of influenza A viruses, H1N1/PR8/34, H3N2 Aichi/2/68, nH1N1/09 were in the range of 0.2 to 0.9 μg/ml, and CoV OC43 was inhibited with an IC_50_ of 0.33 μg/ml. In vitro data on influenza virus are supported by a set of in vivo data with influenza A H1N1/PR8/34 infected mice. In the placebo group only 10% of the animals survived the lethal infection, while in the group treated intranasally with 1.2 mg/ml iota-carrageenan 70% survived when treatment started at the day of infection. When treatment started 48 hours after infection, still 50% of the animals survived. This result was strengthened by the observation that nasal and lung titers were strongly reduced 120 hours after infection similarly as in those animals treated with oseltamivir [8].

Nasal sprays containing 1.2 mg/ml iota-carrageenan have been tested clinically in children and adults suffering from early symptoms of common cold. The spray was applied three times daily for four or seven consecutive days. The patients were tested for the presence of viral pathogens including human rhinoviruses, human coronaviruses (OC43 and 229E), respiratory syncytial virus, metapneumovirus, human influenza A and B strains, as well as parainfluenzaviruses 1–3 before and during treatment (days 1 and 3–5). In one clinical study with adults an additional test for viruses was performed on day 10–11 [13]. The most abundant viral pathogens in both studies were human rhinoviruses, coronaviruses, and influenza A viruses, with 59%, 34.6%, and 18.5%, respectively. The rate of virus-positive subjects was 89% for children from the age of 1–18 years, while in adults the rate was 58%. While in adults only in 15% of the patients more than one viral pathogen was detected, in children this rate increased to 41%. Five different viruses were detected in one child, hCoV OC43, human metapneumovirus, human rhinovirus, influenza B, and parainfluenzavirus 3. As a result of treatment with iota-carrageenan in all clinical studies viral titers were strongly reduced compared to placebo [6]. This reduction of viral titers translated into reduced severity and duration of common cold symptoms and reduction of relapses during the observation period of up to 21 days. The finding that patients may suffer from more than one viral infection also implicates that a pan-anti-viral treatment may be superior compared to virus-specific therapies.

SARS-CoV-2 is inhibited by iota-carrageenan in vitro to the same extent as other respiratory viruses for which a clinical benefit has been proven. Furthermore, iota-carrageenan shows an equivalent effectivity against SARS-CoV-2 as a neutralizing antiserum and soluble ACE2 receptors [20], both well accepted parameters for clinical performance. Therefore, our data suggest that treatment with iota-carrageenan either prophylactically or therapeutically may be similarly effective in humans suffering from COVID-19. Clinical data and post market surveillance data showed that iota-carrageenan is well-tolerated and the number of reported adverse events are very low (1.07 reported adverse events per 100.000 sold units since 2013; more than 8 million units sold). Also in case of the emergence of another “new” respiratory virus, that then again may result in pandemic, iota-carrageenan may serve as a first broadly active treatment to close the gap between virus identification and successful developments of vaccines or specific antiviral medication.

## Supporting information

S1 Fig (PDF)

S2 Fig (PDF)

S3 Fig (PDF)

S4 Fig The original data of the reduction of SARS-CoV-2 viral RNA copy number by iota-carrageenan related to Fig 4 B(A-D) and Table 2 (E). (PDF)

S5 Fig The original data of the reduction of SARS-CoV-2 viral protein (NP) by iota-carrageenan related to Fig 4 B(A-D) and Table 2 (E). (PDF)

S6 Fig The original data of the toxicity of iota-carrageenan on Vero B4 cells related to Fig 5 (A) Raw data with untreated control set to 100% (B) Average of 3 independent experiments.
(PDF)

S7 Fig Raw data in table for SSPL, SARS-CoV-2 (Westernblot), and SARS-CoV-2 (PCR) are integrated into S5 and S4 Figs. (PDF)
