# Supplementary material for: Iota-carrageenan neutralizes SARS-CoV-2 and inhibits viral replication in vitro
Source: PLoS One. 2021 Feb 17;16(2):e0237480. doi: 10.1371/journal.pone.0237480 (PMC7888609; doi:10.1371/journal.pone.0237480)
Supplement: S1 Fig — (PDF) [file pone.0237480.s001.pdf]

S1_Fig1_final

**Raw data**	experiment 1	experiment 2	experiment 3
negative control	353	321	302
positive control	341508	336409	356675
iota-carrageenan	58283	44917	44541
positive serum	34873	40506	35628
negative serum	174192	215626	263060

**Normalized relative values**	%
negative control	0.119
positive control	100
iota-carrageenan	17.521
positive serum	14.002
negative serum	79.322
