# Supplementary material for: Iota-carrageenan neutralizes SARS-CoV-2 and inhibits viral replication in vitro
Source: PLoS One. 2021 Feb 17;16(2):e0237480. doi: 10.1371/journal.pone.0237480 (PMC7888609; doi:10.1371/journal.pone.0237480)
Supplement: S2 Fig — (PDF) [file pone.0237480.s002.pdf]

S2_Fig2_final

**Raw data**	experiment 1	experiment 2	experiment 3	experiment 4	experiment 5	experiment 6
negative control	298	327	427			
positive control	57539	336409	356675	56640	49316	46937
iota-carrageenan 100 µg/ml	9606	4209	4641			
iota-carrageenan 33 µg/ml	4583	10840	9001			
iota-carrageenan 10 µg/ml	11841	12407	8481			
iota-carrageenan 3.3 µg/ml	26152	19804	18955			
iota-carrageenan 1 µg/ml	47607	43362	41127			
iota-carrageenan 0.33 µg/ml	60029	49171	42285			

**Normalized relative values**	%
negative control	0
positive control	100
iota-carrageenan 100 µg/ml	11.2111
iota-carrageenan 33 µg/ml	16.9551
iota-carrageenan 10 µg/ml	19.3043
iota-carrageenan 3.3 µg/ml	41.2743
iota-carrageenan 1 µg/ml	78.9695
iota-carrageenan 0.33 µg/ml	88.8520
