# Supplementary material for: Iota-carrageenan neutralizes SARS-CoV-2 and inhibits viral replication in vitro
Source: PLoS One. 2021 Feb 17;16(2):e0237480. doi: 10.1371/journal.pone.0237480 (PMC7888609; doi:10.1371/journal.pone.0237480)
Supplement: S3 Fig — (PDF) [file pone.0237480.s003.pdf]

S3_Fig3_final

**Raw data**	experiment 1	experiment 2	experiment 3	experiment 4	experiment 5	experiment 6
negative control	298	327	427			
positive control	57539	60358	53230	56640	49316	46937
iota-carrageenan 100 µg/ml	9606	4209	4641			
iota-carrageenan 10 µg/ml	11841	12407	8481			
kappa-carrageenan 100 µg/ml	8688	15197	13010			
kappa-carrageenan 10 µg/ml	39523	32533	34596			
Fuc. U.p. 100 µg/ml	36097	35557	34647			
Fuc. U.p. 10 µg/ml	47014	41098	39751			
CMC 100 µg/ml	44998	39019	30523			
HPMC 100 µg/ml	50190	38436	35320			
Gal-4-SO4 100 µg/ml	52763	54605	48583			
negative control	900	624	450			
positive control	76976	77475	82829	81236	95552	87375
lambda-carrageenan 100 µg/ml	17056	21590	20210			
lambda-carrageenan 10 µg/ml	43301	56655	44895			
Fuc. F.v.100 µg/ml	36354	53291	39806			
Fuc. F.v.10 µg/ml	51225	60799	53998			

**Normalized relative values**	%
negative control	0
positive control	100
iota-carrageenan 100 µg/ml	13.9528
iota-carrageenan 10 µg/ml	21.4936
kappa-carrageenan 100 µg/ml	26.0188
kappa-carrageenan 10 µg/ml	68.1859
Fuc. U.p. 100 µg/ml	71.7140
Fuc. U.p. 10 µg/ml	86.7733
CMC 100 µg/ml	68.1759

HPMC 100 µg/ml	79.6628
Gal-4-SO4 100 µg/ml	82.5848
negative control	0
positive control	100
lambda-carrageenan 100 µg/ml	22.8674
lambda-carrageenan 10 µg/ml	57.4386
Fuc. F.v.100 µg/ml	51.2476
Fuc. F.v.10 µg/ml	65.9496
