# Supplementary material for: Iota-carrageenan neutralizes SARS-CoV-2 and inhibits viral replication in vitro
Source: PLoS One. 2021 Feb 17;16(2):e0237480. doi: 10.1371/journal.pone.0237480 (PMC7888609; doi:10.1371/journal.pone.0237480)
Supplement: S4 Fig — (PDF) [file pone.0237480.s004.pdf]

S4 Figure 4A and Table 2

(A) Experiment 1

Concentration iota-carrageenan (µg/ml)	Viral protein	Viral protein as % of untreated
(untreated) 0 µg/ml	1448704,62	100,00
3,75 µg/ml	84735,66	5,85
7,5 µg/ml	136447,57	9,42
37,5 µg/ml	28451,97	1,96
75 µg/ml	11669,61	0,81

(B) Experiment 2

Concentration iota-carrageenan (µg/ml)	Viral protein	Viral protein as % of untreated
(untreated) 0 µg/ml	948996,09	100,00
3,75 µg/ml	57691,82	6,08
7,5 µg/ml	86764,64	9,14
37,5 µg/ml	-44333,55	0,00
75 µg/ml	-52459,73	0,00

(C) Experiment 3

Concentration iota-carrageenan (µg/ml)	Viral protein	Viral protein as % of untreated
(untreated) 0 µg/ml	251259,60	100,00
3,75 µg/ml	32152,34	12,80
7,5 µg/ml	-28784,99	0,00
37,5 µg/ml	20678,12	8,23
75 µg/ml	32041,81	12,75

(D) Average of the three independent experiments (%).

Concentration Iota-carrageenan (µg/ml)	Average % viral protein reduction (N=3)	Standard deviation
(untreated) 0 µg/ml	100,00	0
3,75 µg/ml	8,24	3,95
7,5 µg/ml	6,19	5,36
37,5 µg/ml	3,40	4,30
75 µg/ml	4,52	7,14

(E) Calculation of IC_{50} and Convidence Interval based on (D)

IC_{50}	Upper 95 % confidence interval	Lower 95 % confidence interval
1,54	2.0259	1,0452

S5 Figure:

The original data of the reduction of SARS-CoV-2 viral protein (NP) by iota-carrageenan related to Figure 4 B (A -D) and Table 2 (E).
