# Supplementary material for: Iota-carrageenan neutralizes SARS-CoV-2 and inhibits viral replication in vitro
Source: PLoS One. 2021 Feb 17;16(2):e0237480. doi: 10.1371/journal.pone.0237480 (PMC7888609; doi:10.1371/journal.pone.0237480)
Supplement: S5 Fig — (PDF) [file pone.0237480.s005.pdf]

S5 Figure 4B and Table 2

(A) Experiment 1

Concentration iota-carrageenan (µg/ml)	RNA copies 1	RNA copies 2	MW (RNA copies 1 und RNA copies 2)	RNA copies as % of untreated
0 µg/ml	1357476,50	1380222,75	1368849,63	100,00
3,75 µg/ml	47958,42	52857,27	50407,84	3,68
7,5 µg/ml	15094,15	15729,43	15411,79	1,13
37,5 µg/ml	3098,96	3838,68	3468,82	0,25
75 µg/ml	5566,67	5232,05	5399,36	0,39

(B) Experiment 2

Concentration iota-carrageenan (µg/ml)	RNA copies 1	RNA copies 2	MW (RNA copies 1 und RNA copies 2)	RNA copies as % of untreated
0 µg/ml	973357,13	972236,44	972796,78	100,00
3,75 µg/ml	6474,80	25632,62	16053,71	1,65
7,5 µg/ml	6691,26	5863,20	6277,23	0,65
37,5 µg/ml	4033,56	4045,49	4039,53	0,42
75 µg/ml	3441,48	3391,41	3416,44	0,35

(C) Experiment 3

Concentration iota-carrageenan (µg/ml)	RNA copies 1	RNA copies 2	MW (RNA copies 1 und RNA copies 2)	RNA copies as % of untreated
0 µg/ml	323516,69	310431,19	316973,94	100,00
3,75 µg/ml	77383,48	72176,66	74780,07	23,59
7,5 µg/ml	12099,64	11074,50	11587,07	3,66
37,5 µg/ml	2641,06	2512,82	2576,94	0,81
75 µg/ml	134,52	1754,97	944,75	0,30

(D) Average of the three independent experiments (%).

Concentration Iota-carrageenan (µg/ml)	Average % RNA copy reduction	Standard deviation
0 µg/ml	100,00	0
3,75 µg/ml	9,64	12,124
7,5 µg/ml	1,81	1,618
37,5 µg/ml	0,49	0,288
75 µg/ml	0,35	0,0483

(E) Calculation of IC_{50} and Convidence Interval based on (D)

IC_{50}	Upper 95 % confidence interval	Lower 95 % confidence interval
0,0464	0,1835	-0,0908

S4 Figure 4A:

The original data of the reduction of SARS-CoV-2 viral RNA copy number by iota-carrageenan related to Figure 4 B (A -D) and Table 2 (E).
