# Supplementary material for: Iota-carrageenan neutralizes SARS-CoV-2 and inhibits viral replication in vitro
Source: PLoS One. 2021 Feb 17;16(2):e0237480. doi: 10.1371/journal.pone.0237480 (PMC7888609; doi:10.1371/journal.pone.0237480)
Supplement: S6 Fig — The original data of the toxicity of iota-carrageenan on Vero B4 cells related to Fig 5 (A) Raw data with untreated control set to 100% (B) Average of 3 independent experiments. (PDF) [file pone.0237480.s006.pdf]

S6 Figure 5 and Table 2

(A) Raw data. Extinkition of untreated control was set to 100 %.

Experiment #	untreated	Iota-carrageenan (µg/ml)					Staurosporin [M]
		0,01	0,1	1,0	10,0	100,0	
1	100	96,37	94,38	100,65	110,13	128,26	60,84
2	100	83,47	83,03	88,95	100,14	107,18	23,42
3	100	83,65	82,13	79,54	93,20	118,82	25,06

(B) Average of the three independent experiments (%).

Experiment #	% of untreated control	Iota-carrageenan (µg/ml)					Staurosporin [M]
		0,01	0,1	1,0	10,0	100,0	
Average (Exp. 1 – 3)	100	87,83	86,51	89,71	101,16	118,09	36,44
Standard deviation	0	7,40	6,83	10,58	8,51	10,56	21,15

S6 Figure 5:

The original data of the toxicity of iota-carrageenan on Vero B4 cells related to Figure 5 (A) Raw data with untreated control set to 100 % (B) Average of 3 independent experiments
