# Supplementary material for: Iota-carrageenan neutralizes SARS-CoV-2 and inhibits viral replication in vitro
Source: PLoS One. 2021 Feb 17;16(2):e0237480. doi: 10.1371/journal.pone.0237480 (PMC7888609; doi:10.1371/journal.pone.0237480)
Supplement: S7 Fig — (PDF) [file pone.0237480.s007.pdf]

S7_tab2_final

**Raw data**	exp.1	exp.2	exp. 3	exp. 4	exp.t 5	exp.6	exp.7	exp. 8	exp. 9	exp. 10
negative control	0.326	0.265	0.132	0.197	0.226					
positive control	1.019	1.121	0.998	0.991	1.169	0.917	1.131	1.207	1.010	0.944
iota-carrageenan 100 µg/ml	0.275	0.279	0.244	0.236	0.329					
iota-carrageenan 3 µg/ml	0.372	0.277	0.315	0.300	0.270					
iota-carrageenan 9 µg/ml	0.391	0.250	0.252	0.315	0.291					
iota-carrageenan 2.7 µg/ml	0.289	0.324	0.294	0.293	0.334					
iota-carrageenan 0.8 µg/ml	0.578	0.863	0.480	1.279	0.436					
iota-carrageenan 0.24 µg/ml	0.453	0.523	0.506	0.909	0.766					
iota-carrageenan 0.07 µg/ml	0.460	0.684	0.623	1.221	0.712					
iota-carrageenan 0.02 µg/ml	0.662	0.988	0.937	1.000	1.157					
iota-carrageenan 0.007 µg/ml	0.691	0.752	0.978	1.270	0.996					

**Normalized relative values**	%
negative control	0
positive control	100
iota-carrageenan 100 µg/ml	5.2830
iota-carrageenan 3 µg/ml	9.4461
iota-carrageenan 9 µg/ml	8.5940
iota-carrageenan 2.7 µg/ml	9.4461
iota-carrageenan 0.8 µg/ml	60.6208
iota-carrageenan 0.24 µg/ml	48.9592
iota-carrageenan 0.07 µg/ml	62.1789
iota-carrageenan 0.02 µg/ml	87.5959
iota-carrageenan 0.007 µg/ml	86.2082

Raw data in table for SSPL, SARS-CoV-2 (Westernblot), and SARS-CoV-2 (PCR) are integrated into S4 Fig4a_Tab 2_final and S5 Fig4b_Tab2_final.
